# A web-based integrative transcriptome analysis, RNAseqChef, uncovers the cell/tissue type-dependent action of sulforaphane

**DOI:** 10.1016/j.jbc.2023.104810

**Published:** 2023-05-11

**Authors:** Kan Etoh, Mitsuyoshi Nakao

**Affiliations:** Department of Medical Cell Biology, Institute of Molecular Embryology and Genetics, Kumamoto University, Kumamoto, Japan

**Keywords:** RNAseqChef, open resource, transcriptomics, sulforaphane, NRF2 (*NFE2L2*), antioxidant response, ATF6, unfolded protein response

## Abstract

RNA sequencing (RNA-seq) is a powerful technique for understanding cellular state and dynamics. However, comprehensive transcriptomic characterization of multiple RNA-seq datasets is laborious without bioinformatics training and skills. To remove the barriers to sequence data analysis in the research community, we have developed “RNAseqChef” (RNA-seq data controller highlighting expression features), a web-based platform of systematic transcriptome analysis that can automatically detect, integrate, and visualize differentially expressed genes and their biological functions. To validate its versatile performance, we examined the pharmacological action of sulforaphane (SFN), a natural isothiocyanate, on various types of cells and mouse tissues using multiple datasets *in vitro* and *in vivo*. Notably, SFN treatment upregulated the ATF6-mediated unfolded protein response in the liver and the NRF2-mediated antioxidant response in the skeletal muscle of diet-induced obese mice. In contrast, the commonly downregulated pathways included collagen synthesis and circadian rhythms in the tissues tested. On the server of RNAseqChef, we simply evaluated and visualized all analyzing data and discovered the NRF2-independent action of SFN. Collectively, RNAseqChef provides an easy-to-use open resource that identifies context-dependent transcriptomic features and standardizes data assessment.

RNA sequencing (RNA-seq) is a core technology used for comprehensive gene expression analyses (1). RNA-seq datasets are deposited in public databases such as the Gene Expression Omnibus (GEO) so that researchers can freely utilize the available data (2). Currently, more than 100,000 datasets derived from cell lines and tissues of various species are available for re-analyses, which are useful resources for those with a general interest in the life science field. However, a series of data investigations, including differentially expressed gene (DEG) analysis, data integration, clustering, functional analysis, and visualization, are considerably laborious for most researchers and scientists not specializing in bioinformatics.

Sulforaphane (SFN) is a natural isothiocyanate generated by cruciferous vegetables such as broccoli. SFN is known to interact with the active cysteine residues of KEAP1 (Kelch-like ECH-associated protein 1), resulting in the activation of nucleus factor-E2-related factor 2, NRF2 (*NFE2L2*), which is the master transcription factor for redox homeostasis (3, 4, 5). SFN treatment has anti-obesity and antidiabetic effects through NRF2 activation in multiple models, such as diet-induced obese mice and rodents (6, 7, 8). Indeed, SFN-induced activation of NRF2 has been shown to ameliorate oxidative and endoplasmic reticulum (ER) stress, which causes excessive adipogenesis, lipid accumulation, inflammation, and fibrosis (9, 10, 11, 12). However, recent studies have shown the cytotoxic effect of SFN in cancer cells *via* mitochondrial damage and apoptosis induction (13, 14). Although it is plausible that SFN actions depend on cellular status, little is known about the context-dependent effects of SFN. Furthermore, the NRF2-independent actions of SFN remain poorly understood.

Upon RNA-seq analysis of multiple datasets, numerous workflows are required for the user’s demands. However, existing web platforms are adjusted for the analysis of “single dataset” (controls *versus* subjects) but not “multiple datasets” ([controls *versus* subjects] × n). In this study, we developed RNAseqChef (an RNA-seq data controller highlighting expression features), a web-based systematic analysis of multiple RNA-seq datasets. Using this platform, we performed integrative transcriptome analysis of SFN-treated cultured cells and model mice. We found that SFN-induced ATF6-mediated unfolded protein response (UPR) without NRF2 activation, and that SFN-induced UPR occurred in the liver, but not in other metabolic tissues, in diet-induced obese mice. We also found that SFN treatment downregulated the expression of collagen and circadian rhythm-associated genes involved in fibrosis and lipid metabolism, respectively. Thus, SFN-induced transcriptomic changes depended on the cell/tissue type. RNAseqChef platform evaluates molecular features without special bioinformatic skills in biological and medical research fields and allows integration, simplification, user-centered, and standardization of data assessment.

## Results

### Design of RNAseqChef

**Figure 1:**
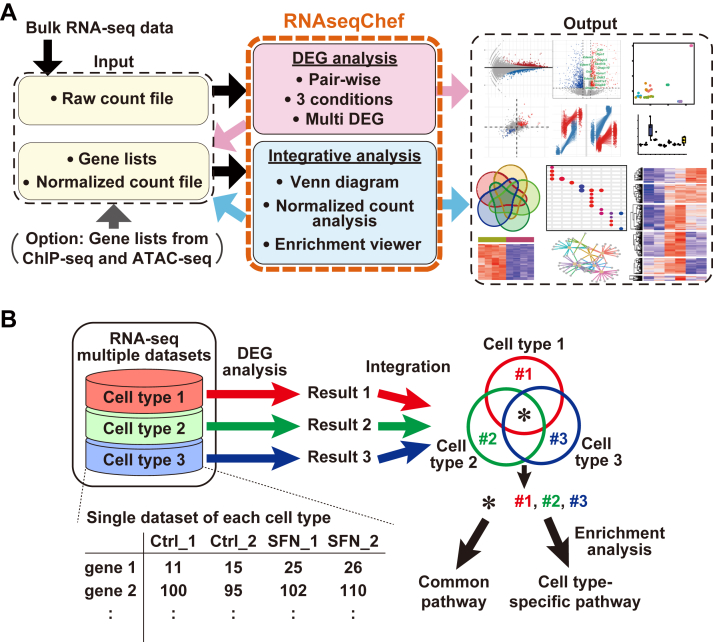
**Overview of RNAseqChef (RNA-seq data controller highlighting gene expression feature).***A*, RNAseqChef is a web-based platform of systematic transcriptome analysis and can automatically detect, integrate, and visualize the DEGs and their biological functions, by uploading the raw count files of interest. The “DEG Analysis Unit” consists of “Pair-wise DEG”, “3 conditions DEG”, and “Multi DEG”. The “Integrative Analysis Unit” then consists of “Venn diagram,” “Normalized count analysis,” and “Enrichment viewer.” The result files obtained from the DEG Analysis Unit can be used as input files for the Integrative Analysis Unit, which makes it easy to perform the bioinformatic studies on demand. *B*, analysis of the common and cell/tissue-specific effects of SFN on transcriptome using RNAseqChef. DEG, differentially expressed gene; SFN, Sulforaphane.

**Figure 2:**
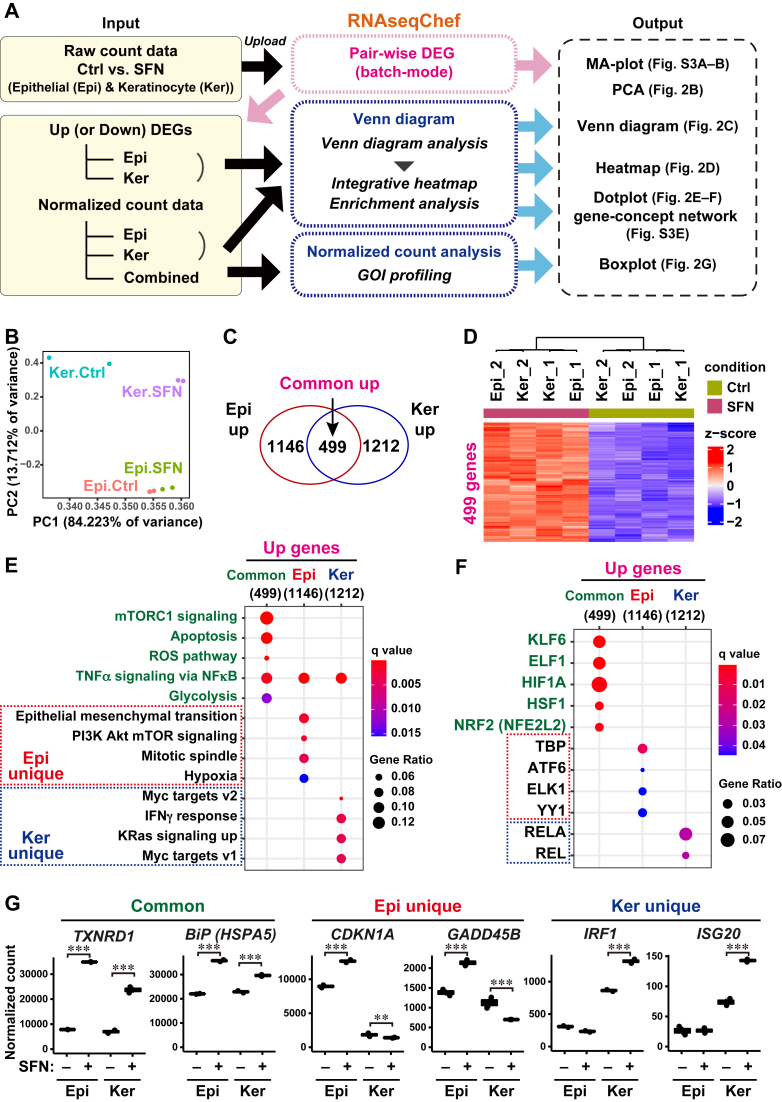
**Cellular response to SFN is different among cell types.***A*, schematic representation of the RNAseqChef analysis designed to highlight SFN-induced transcriptomic features and their cell-type dependency. Raw count data were obtained from public RNA-seq datasets of epithelial cells (Epi; GSE141740) and HaCaT keratinocytes (Ker; GSE185320), under control (Ctrl; DMSO-treated) and 10 μM SFN-treated conditions (each n = 2). Along with the workflow in Figure 1, *A* and *B*, it takes approximately 5 min to complete all analyzing steps. *B*, transcriptome-based PCA of Ctrl and SFN-treated cells. *C*, Venn diagram of genes significantly upregulated by SFN treatment (using DESeq2 (FDR < 0.01)). *D*, heatmap of commonly upregulated genes shown in (*C*). *E* and *F*, top-ranked functional pathways (*E*) and transcription factors (TFs) (*F*) were enriched in gene sets upregulated by SFN treatment, including 499 common genes (*green*), 1146 Epi unique genes (*red*), and 1212 Ker unique genes (*blue*), as shown in (*C*). Enrichment analysis was performed based on MSigDB hallmark (*E*) and DoRothEA regulon gene set (*F*). FDR <0.05. *G*, normalized expression values of the representatives for common, Epi unique, and Ker unique genes upregulated by SFN. ∗∗*p* < 0.01; ∗∗∗*p* < 0.001. FDR, false discovery rate; PCA, Principal component analysis; SFN, Sulforaphane.

RNAseqChef (https://imeg-ku.shinyapps.io/RNAseqChef/) is a user-centered web-based application for the systematic integrative analysis of RNA-seq data (Figs. 1*A* and S1; Movie S1). The user’s graphical interface of RNAseqChef comprises “Menu”, “Settings”, “Output” and “Tab” panels, which do not require any special bioinformatics skills to navigate (Fig. S2). In contrast to the existing web platforms (15, 16, 17), users can analyze not only a single dataset but also integrate and evaluate multiple datasets (Fig. 1*B*). To establish the versatility of RNAseqChef, we divided its core functions into six sections, which enabled us to handle various types of input files (Fig. 1*A*). RNAseqChef consists of the following 2 units: DEG Analysis Unit (“Pair-wise”, “3 conditions DEG” and “Multi DEG”) and Integrative Analysis Unit (“Venn diagram”, “Normalized count analysis” and “Enrichment viewer”). The DEG Analysis Unit was optimized for the analysis of a single dataset to automatically perform DEG detection, pathway analysis, and data visualization by uploading the raw count data. “Pair-wise DEG” was adjusted for pair-wise comparison analysis, and “3 conditions DEG” was enhanced for multiple comparison analysis of three groups using EBSeq (18). “Multi DEG” was optimized for the analysis of time-series experiments using the DESeq2 likelihood ratio test (19). To increase the throughput for the pairwise analysis of multiple datasets and subsequent integrative analysis, we created the batch-mode in “Pair-wise DEG”, which sequentially performs DEG analysis of all uploaded files, and then outputs the following results: clustering plot, MA-plot, DEG result, lists of both upregulated and downregulated DEGs, and normalized count data. The results files obtained from the DEG Analysis Unit can be used as input files for the Integrative Analysis Unit, which makes it easy to perform a flexible analysis on demand (*e.g.*, Fig. 2*A* and Movie S2). “Venn diagram” was optimized for Venn diagram and subsequent analysis (heatmap and pathway analysis). “Normalized count analysis” was adjusted for clustering analysis of normalized count data, and “Enrichment viewer” was optimized for functional pathway and promoter motif analysis. In addition, the pathway databases that are used for enrichment analysis in RNAseqChef can be referred to from the “MSigDB” (Molecular Signatures Database) and “DoRothEA regulon” in the “More” section of RNAseqChef.

### Biological responses to SFN treatment depend on cell types

Based on our criteria that the read depth should be more than 10 million reads per sample, we found reliable *in vitro* datasets of cultured human cells, epithelial cells (Epi: GSE141740), and keratinocytes (Ker: GSE185320), which were treated with control (Ctrl) or SFN (12, 20). To characterize the transcriptomic features in response to SFN, we performed an integrative analysis of these datasets using RNAseqChef (Fig. 2*A*). It took approximately 5 min to complete all steps (Movie S2). Principal component analysis (PCA) showed that the cell type datasets (Epi or Ker) with treatment (Ctrl or SFN) were distinctly positioned (Fig. 2*B*). To identify the common and cell type-specific genes affected by SFN treatment, we detected DEGs from each dataset using DESeq2 (Fig. S3, *A* and *B*) and conducted Venn diagram analysis (Figs. 2*C* and S3*F*). SFN treatment upregulated 499 genes in both cell types (Fig 2, *C* and *D*). In contrast, 1146 and 1212 genes were upregulated in SFN-treated Epi and Ker, respectively (Fig. 2*C*). To clarify the biological relevance of SFN-associated genes, we performed enrichment analysis based on the MSigDB hallmark gene set (Figs. 2*E* and S3, *C* and *D*). Consistent with previous reports (5, 21, 22), the top-ranked pathways related to the 499 commonly upregulated genes were the mammalian target of rapamycin complex 1 (mTORC1) signaling, apoptosis, reactive oxygen species (ROS) pathway, and glycolysis (Fig. 2*E*). In addition, transcription factor (TF) enrichment analysis based on the DoRothEA regulon gene set identified a well-known SFN-target NRF2, which transcriptionally activates antioxidant genes (*TXNRD1, HMOX1,* and *NQO1*) (Figs. 2*F* and S3*E*). In contrast, 1212 Ker-specific genes, but not 1146 Epi-specific genes, enriched inflammation-related pathways, such as interferon-γ (IFNγ) response (*IRF1* and *ISG20*), and TFs, such as RELA (targeting *IRF1*) (Fig. 2, *E* and *G*). We also analyzed downregulated genes in SFN-treated cells using enrichment analysis (Fig. S3*F*). The 242 commonly downregulated genes by SFN-enriched TFDP1/E2F targets (*GINS1* and *SRSF2*) (Fig. S3, *G*–*I*). A total of 1652 Ker-specific genes, but not Epi-specific genes, enriched p53 pathway (*CDKN1A*) (Figs. 2*G* and S3, *G* and *H*) and estrogen response (*KRT13* and *KRT15*) (Fig. S3, *G*–*I*). Thus, we found that SFN has both common and cell type-specific effects using RNAseqChef analysis of multiple RNA-seq datasets.

### SFN induces the unfolded protein response in an NRF2-independent manner

**Figure 3:**
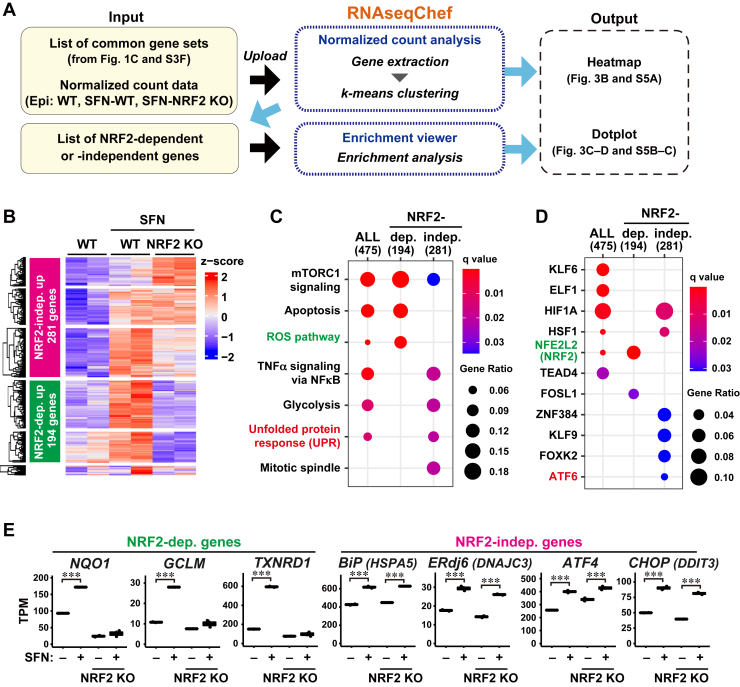
**SFN induces the unfolded protein response in an NRF2-independent manner.***A*, schematic representation of the RNAseqChef analysis designed to identify the NRF2-dependent or independent actions of SFN in epithelial cells. Gene extraction of the common genes from the normalized count data (control WT, SFN-treated WT and NRF2 KO, each n = 2) was performed in the “Normalized count analysis” section. Subsequent k-means clustering was done using the tab panel named “k-means clustering” in the same section. Enrichment analysis was performed by uploading the result file obtained from k-means clustering in the “Enrichment viewer” section. *B*, the k-means clustering approach separated 499 commonly upregulated genes (shown in Fig. 2*C*) into two groups based on NRF2 dependency. The *magenta* and *green box* indicate 281 NRF-independent and 194 NRF2-dependent upregulated genes, respectively. The 24 genes (in the *bottom* cluster of the heatmap) were not separated into the above groups because their expression patterns were not obvious. *C* and *D*, top-ranked functional pathways (*C*) and TFs (*D*) were enriched in gene sets upregulated by SFN treatment, including 475 ALL genes (sum of the following genes), 194 NRF2-dependent genes, and 281 NRF2-independent genes. Enrichment analysis was performed based on MSigDB hallmark (*C*) and DoRothEA regulon gene set (*D*). FDR <0.05. *E*, TPM expression values of the representative NRF2-dependent and NRF2-independent genes that were upregulated by SFN. ∗∗∗*p* < 0.001. FDR, false discovery rate; NRF2, nucleus factor-E2-related factor 2; SFN, Sulforaphane; TPM, Transcripts per million; WT, wild type.

SFN has been reported to trigger an oxidative stress response through the activation of NRF2 (3, 23), but its NRF2-independent functions remain poorly understood. To address this, we performed a clustering analysis of genes commonly upregulated by SFN (Figs. 2*C* and 3*A*). For this purpose, we used normalized count data from control WT, SFN-treated WT, and SFN-treated NRF KO epithelial cells (GSE141740) for the clustering approach. NRF2 dependency was defined as whether NRF2 KO canceled the effects of SFN on gene expression. k-means clustering analysis detected 194 NRF2-dependent and 281 NRF2-independent upregulated genes (Fig. 3*B*). As expected, enrichment analysis showed that NRF2-dependent upregulated genes enriched both the ROS pathway and the NRF2 targets (such as *NQO1*, *GCLM*, and *TXNRD1*), while NRF2-independent upregulated genes did not at all (Fig. 3, *C*–*E*), indicating that RNAseqChef had a secure clustering capacity. In contrast, NRF2-independent upregulated genes enriched the UPR and its regulator ATF6 targets (such as *BiP* (binding immunoglobulin protein, gene symbol *HSPA5*) and *ERdj6* (ER-resident protein, gene symbol *DNAJC3*)) (Fig. 3, *C*–*E*). Furthermore, similar results were obtained by EBSeq multiple comparison analyses of the three groups (control WT, SFN-WT, and SFN-NRF2 KO) by “3 conditions DEG” in RNAseqChef (Fig. S4*A* and Movie S3). We detected 293 NRF2-dependent upregulated genes that enriched the ROS pathway (*q* value: 1.1 × 10^−4^) and NRF2 targets (*q* value: 5.8 × 10^−3^) (Fig. S4, *B*–*E*). We also detected 578 NRF2-independent upregulated genes that enriched UPR (*q* value: 1.4 × 10^−3^) and ATF6 targets (*q* value: 6.5 × 10^−3^) (Fig. S4, *F*–*I*). We then performed a clustering analysis of 242 genes commonly downregulated by SFN and detected 91 NRF2-dependent and 151 NRF2-independent downregulated genes (Figs. S3*F* and S5*A*). NRF2-dependent downregulated genes enriched E2F targets and the TGFβ signaling pathway (Fig. S5, *B* and *C*), but NRF2-independent downregulated genes did not.

UPR is induced by ER stress to attenuate protein synthesis, increase protein folding, and enhance the activity of protein degradation pathways, which are important for preventing pathological conditions such as amyloid disease and diabetes (24, 25). ATF6 is an ER stress sensor for the UPR that upregulates cytoprotective pathways, including protein folding and ER homeostasis (26). Although it has previously been shown that SFN modulates UPR through an NRF2-dependent mechanism (10, 27), it is unknown whether SFN enhances UPR without NRF2. To further examine the effect of NRF2 on UPR, we analyzed the RNA-seq data of constitutively active NRF2 (caNRF2)-expressing fibroblasts (GSE106097) (28). NRF2-dependent genes (*Nqo1* and *Gclm*), but not UPR genes (*Atf4*, *BiP*, and *ERdj6*), were significantly upregulated in caNRF2-expressed cells (Fig. S6*A*). Further, we examined the recent RNA-seq data of human uterine leiomyosarcoma SK-UT-1 cells that were treated with 5 μM SFN (GSE205777) (29). Consistent with the results of Epi cells (Fig. 3*E*), both NRF2-target genes and UPR-related genes were upregulated in SFN-treated SK-UT-1 cells (Fig. S6*B*). These results suggested that SFN functions as a UPR inducer in an NRF2-independent manner.

### Upregulated genes by SFN treatment depend on tissue types in diet-induced obese mice

To address SFN action *in vivo*, we analyzed the RNA-seq dataset of five major metabolic tissues, including the liver, skeletal muscle, brown adipose tissue (BAT), epididymal white adipose tissue (eWAT), and inguinal WAT (iWAT), in diet-induced obese mice in the presence or absence of SFN treatment (6). C57BL/6J mice were fed a high-fat diet (HFD, 60% of the calories from fat) for 16 weeks to induce obesity and were treated with daily intraperitoneal SFN injections (5 mg/kg, i.p.) during the last 1 week of the experimental period (Fig. S7*A*). SFN treatment resulted in body weight loss and improvement in obesity, where it was reported that NRF2-dependent antioxidant genes were upregulated in the skeletal muscle and that fatty acid synthesis genes were downregulated in the liver, BAT, and eWAT (6). However, the common and tissue-specific effects of SFN have not yet been explored using unbiased transcriptome analysis.

**Figure 4:**
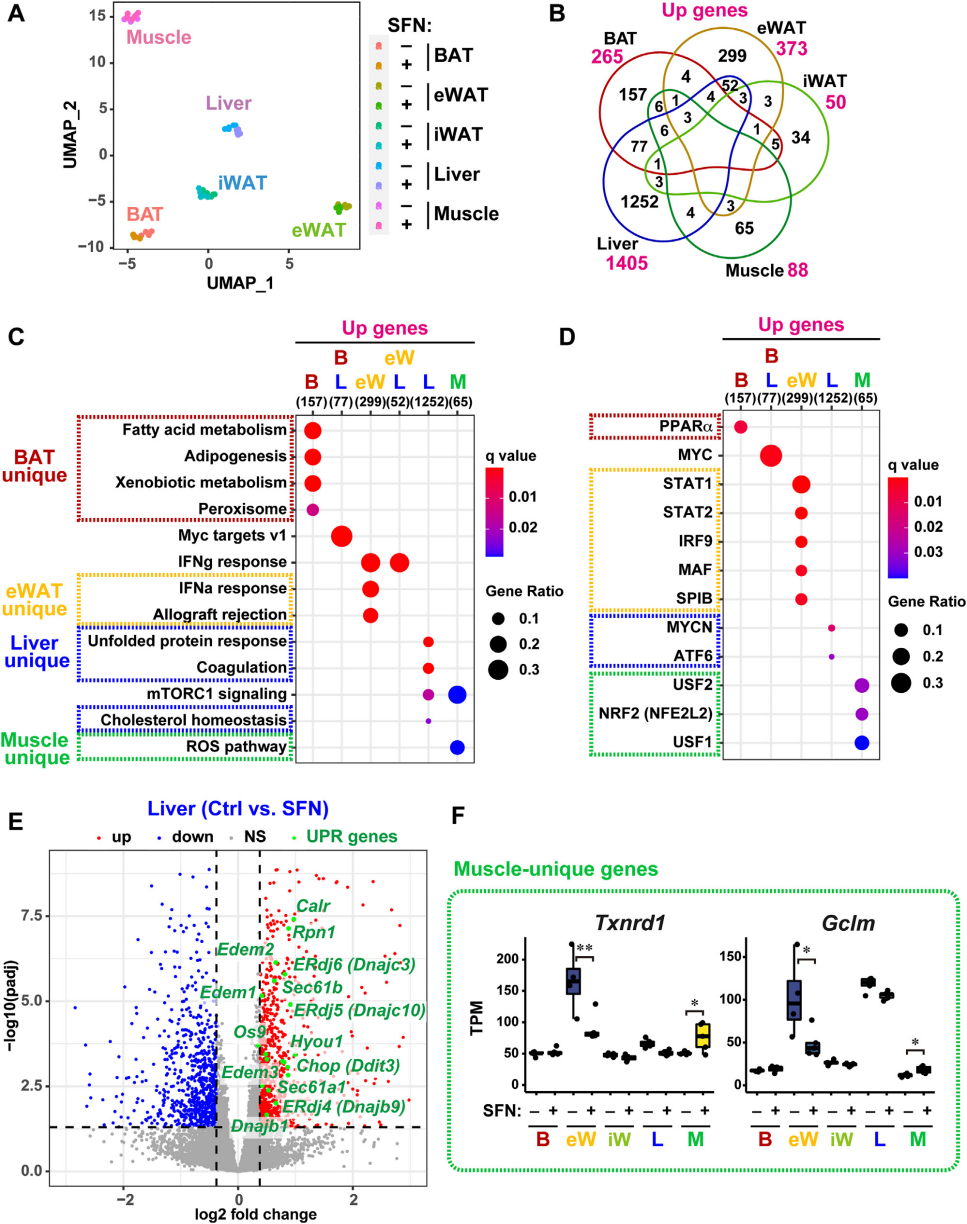
**SFN treatment induces tissue-specific transcriptomic changes in obese mice.***A*, transcriptome-based UMAP of five metabolic tissues such as BAT, eWAT, iWAT, liver, and skeletal muscle in high fat diet-fed obese mice. Data set is GSE181477 (untreated Ctrl (−) and SNF-treated (+); each n = 5, except for eWAT (n = 4)). *B*, Venn diagram of genes significantly upregulated by SFN treatment (FDR < 0.05). *C* and *D*, top-ranked functional pathways (*C*) and TFs (*D*) were enriched in gene sets upregulated by SFN treatment, including 157 BAT unique genes (*red*), 299 eWAT unique genes (*yellow*), 1252 liver unique genes (*blue*) and 65 muscle unique genes (*green*), as shown in (*B*). Enrichment analysis was performed based on MSigDB hallmark (*C*) and DoRothEA regulon gene set (*D*). FDR <0.05. *E*, volcano plot obtained from pair-wise DEG analysis (Ctrl *versus* SFN) of the liver in HFD-fed obese mice. As indicated with *green dots,* ATF6-targeted UPR genes were significantly upregulated by SFN treatment (FDR < 0.05). *F*, TPM expression values of the representative skeletal muscle-unique genes upregulated by SFN. ∗*p* < 0.05; ∗∗*p* < 0.01. BAT, brown adipose tissue; eWAT, epididymal white adipose tissue; FDR, false discovery rate; HFD, high-fat diet; iWAT, inguinal WAT; SFN, Sulforaphane; TPM, Transcripts per million; UPR, unfolded protein response; WT, wild type.

Uniform manifold approximation and projection (UMAP) placed the dataset derived from the same tissue in close proximity (Fig. 4*A*), indicating that each tissue type had different transcriptomic features. To identify the common and tissue-specific genes affected by SFN treatment, we detected DEG genes from each tissue type using DESeq2 and then conducted Venn diagram analysis (Figs. 4*B* and S7, *B* and *C*). Interestingly, there was no overlap of upregulated genes among the five tissues, and most intersections among more than two tissues consisted of fewer than ten genes, suggesting that SFN treatment induced distinct transcriptomic changes depending on the tissue type of HFD-fed obese mice. To gain insight into the biological role of the upregulated genes in each tissue type of SFN-treated HFD-fed mice, we performed an enrichment analysis of the tissue-specific upregulated genes using the MSigDB hallmark and DoRothEA regulon gene sets (Figs. 4, *C*–*F*, and S8, *A*–*C*). The results showed that adipogenesis and its regulator PPARα were upregulated in the BAT; IFNα response and its regulator STAT1 were upregulated in the eWAT; UPR and ATF6 were upregulated in the liver; and ROS pathway and NRF2 were upregulated in the skeletal muscle. In particular, ATF6-targeted genes involved in UPR function, including protein folding (such as *ERdj4/6, DNAJB1*, and *Hyou1*), quality control and degradation (*Edem1/2/3* and *Os9*), and ER protein import (*Sec61a/b*), were upregulated in the liver (Fig. 4*E*), but not in other tissues, in SFN-treated HFD-fed mice. In contrast, muscle-unique upregulated genes included anti-oxidant and NRF2 targets (*Txnrd1* and *Gclm*) (Fig. 4*F*), although the *Nrf2* gene itself was moderately expressed in the muscle (Fig. S8*D*). These data revealed that SFN action significantly depends on the tissue type in HFD-fed obese mice.

### SFN treatment downregulates collagen and circadian rhythm genes in obese mice

**Figure 5:**
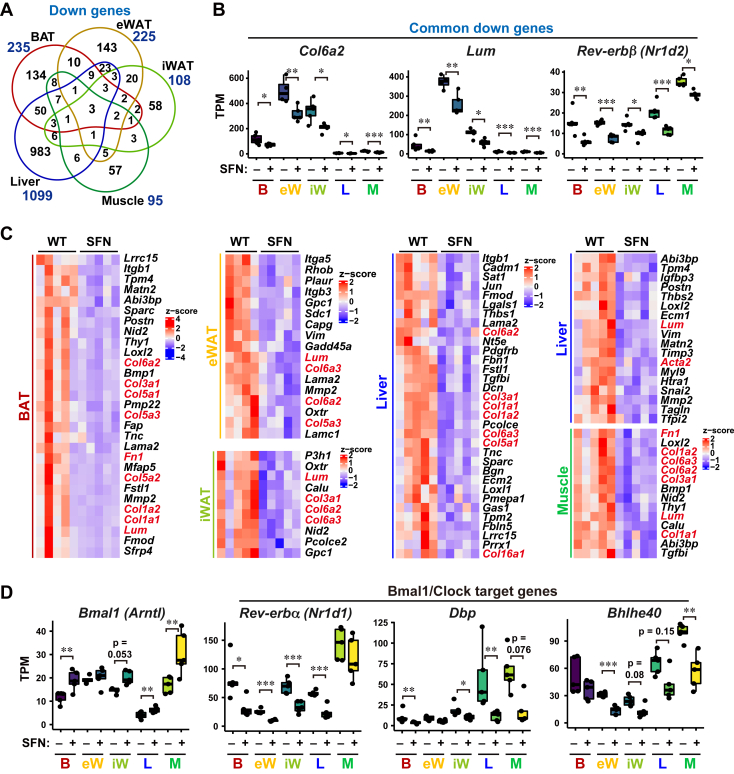
**SFN treatment downregulates collagen genes and BMAL1/CLOCK target genes in obese mice.***A*, Venn diagram of genes significantly downregulated by SFN treatment (FDR < 0.05). *B*, TPM expression values of genes commonly downregulated by SFN in five metabolic tissues of HFD-fed obese mice. ∗*p* < 0.05; ∗∗*p* < 0.01; ∗∗∗*p* < 0.001. *C*, heatmap of the hallmark gene set “Epithelial mesenchymal transition,” which was downregulated in the tissues of SFN-treated obese mice, as shown in Fig. S9. Fibrosis-associated genes are highlighted in *red*. *D*, TPM expression values of *Bmal1* and BMAL1/CLOCK target genes downregulated in multiple tissues of SFN-treated HFD-fed obese mice. ∗*p* < 0.05; ∗∗*p* < 0.01; ∗∗∗*p* < 0.001. FDR, false discovery rate; HFD, high-fat diet; SFN, Sulforaphane; TPM, Transcripts per million.

Finally, we focused on the downregulated genes in SFN-treated HFD-fed obese mice. In contrast to the absence of commonly upregulated genes (Fig. 4*B*), SFN treatment downregulated three genes (*Col6a2*, *Lum*, and *Rev-erbβ* (nuclear receptor subfamily one group D member 1, gene symbol *NR1D1*)) in the five tissues tested (Fig. 5, *A* and *B*). In addition, the epithelial-mesenchymal transition pathway, which contained genes involved in fibrosis, wound healing, and metastasis, was enriched in the commonly downregulated genes among these tissue types (Fig. S9, *A*–*E*). Several genes, including collagen (*Col1a1/2/3*, *Col3a1, Col5a1*, and *Col6a1/2/3*), *Fn1*, and *Lum,* were evidently decreased in the examined tissues of SFN-treated HFD-fed mice (Fig. 5*C*), which has been implicated in tissue fibrosis (30, 31, 32, 33). Furthermore, we detected BMAL1 (brain and muscle ARNT-like 1, gene symbol *Arntl*), a regulator of circadian rhythm, whose targets were enriched in the downregulated genes in BAT, iWAT, and skeletal muscle (Fig. S9, *A*, *C*, and *E*). BMAL1/CLOCK-target genes that are involved in circadian oscillation (such as *Rev-erbα/β*) and clock-controlled energy metabolism genes (*Dbp* and *Bhlhe40*) were downregulated in more than two tissues of SFN-treated HFD-fed obese mice (Fig. 5, *B* and *D*). To exclude the effect of *in vivo* environmental factors on circadian rhythms, we checked the expression levels of circadian genes in SFN-treated cells *in vitro* (Fig. S10). SFN treatment reduced the expression of multiple circadian oscillators (*REV-ERBβ*, *CRY1*, and *PER1/2/3*) in both WT and NRF2 KO epithelial cells, although there were no evident changes in the mRNA levels of *BMAL1* and *CLOCK*. Thus, these results suggest that SFN downregulates collagen synthesis and circadian rhythms in multiple tissues and cultured cells. In addition, as was the unique tissue-type upregulated genes by SFN treatment (Fig 4, *C* and *D*), we found the presence of unique tissue-type downregulated genes (Fig. S11). Taken together, RNAseqChef analysis of multiple RNA-seq datasets demonstrated that SFN had common and tissue-specific effects *in vivo*.

## Discussion

To maximize the comprehensive capacity of RNA-seq analysis in biological research fields, we developed a user-centered web application, RNAseqChef (Fig. 1*A*). The present study verified that RNAseqChef enabled us to perform DEG analysis, data integration, and visualization (Movies S1–S3). As demonstrated in our SFN study, the performance (time) was about 5 min for visualizing all analyzing data (in the case of Fig. 2) (Movie S2). In addition, running RNAseqChef did not depend on the user's computer specs because data analysis was performed on the server. Because the count data of bulk RNA-seq are usually obtained from public databases, such as GREIN (34), anyone can readily perform and reproduce integrative RNA-seq analysis using RNAseqChef. Furthermore, gene lists obtained from the other omics analyses (*e.g.*, ChIP-seq, ATAC-seq, and methylome collected in ChIP-atlas (35)) can be used as an input file for the Integrative Analysis Unit in RNAseqChef to merge RNA-seq data with epigenome information (36) (Fig. 1*A*). Thus, RNAseqChef would facilitate our understanding of the physiological and pathological processes in multiple omics layers.

Previous studies have shown that SFN has an anti-obesity function in an NRF2-dependent manner, but a transcriptomic characterization of SFN action has not been performed in detail. We found that SFN treatment specifically activated ATF6-mediated UPR in the liver of HFD-fed obese mice (Fig. 4, *C*–*E*). It has been reported that ATF6 is important for attenuating hepatic steatosis under ER stress triggers, such as HFD, and that hepatic ATF6-conditional knockout causes lipid accumulation in the liver through the upregulation of the PPARγ pathway that facilitates lipogenesis (37, 38). Accordingly, we speculated that SFN ameliorates hepatic lipid accumulation through an ATF6-dependent UPR. Consistent with this, SFN treatment decreased the expression of a series of genes responsible for acetyl-CoA synthesis (such as *Me1* and *Acly*), fatty acid synthesis from acetyl-CoA (*Acaca* and *Fasn*), fatty acid elongation and desaturation (*Elovl6* and *Scd1*), and lipid deposition (*Pparγ* and *Cidec*) (Fig. S8, *E* and *F*). In addition, SFN treatment affected the expression levels of fibrosis-related collagen and circadian rhythm genes. Previous reports have shown that SFN attenuates hepatic fibrosis *via* the NRF2-mediated inhibition of TGFβ signaling (11). Our RNA-seq analysis consistently showed that SFN treatment downregulated the TGFβ pathway in an NRF2-dependent manner (Fig. S5*B*), suggesting that SFN treatment suppresses the synthesis of profibrogenic proteins in obese mice. The core transcription factor BMAL1 in the circadian rhythm has been shown to regulate energy metabolism (39). We found that SFN treatment in obese mice decreased BMAL1-target genes, such as *Dbp* and *Bhlhe40* (Fig. 5*D*), which are important for the induction of PPARγ and SREBP-1c, which are key transcriptional factors involved in lipid metabolism (40, 41). It is possible that the SFN-mediated regulation of circadian rhythms contributes to the anti-obesity effect.

Our RNA-seq analyses utilized high-quality public RNA-seq datasets from independent research groups and demonstrated the potential action of SFN. To validate our findings in this study, further studies will be needed to understand the contribution of the SFN-induced transcriptome to phenotypic effects and the molecular mechanism of SFN action, using different concentrations of SFN in various cells/tissues tested. Actually, previous reports have shown that SFN has a hormetic dose-dependent effect and exhibits different tissue distribution (42, 43, 44, 45, 46).

RNAseqChef was optimized for analyzing “bulk RNA-seq” count data from public databases or their sequencing results. Users can utilize count data, but not RNA-seq raw data (*i.e.*, fastq files), as input for this application. Thus, in the case that you cannot obtain the count data from the database, you would need to obtain fastq files from the Sequence Read Archive and then perform pre-processing such as quality control and mapping by using a command line or web services such as Galaxy (47). In addition, RNAseqChef is not currently available for “single-cell RNA-seq” analysis. Beyond these issues, as a further advantage of RNAseqChef, the user can simply merge the RNA-seq dataset with epigenomic datasets, such as ChIP-seq and ATAC-seq, for an overview of multilayered omics data. Collectively, RNAseqChef would provide us with sufficient application to investigate RNA-seq datasets from biological and pharmacological effects to achieve standardization of data assessment.

## Experimental procedures

### Public datasets

Raw datasets from public databases were collected from the “GEO repository” (https://www.ncbi.nlm.nih.gov/geo/). The following public datasets regarding the SFN-induced transcriptome were used: 10 μM SFN-treated epithelial cells (GSE141740), 10 μM SFN-treated HaCaT keratinocytes (GSE185320), caNRF2-expressing fibroblasts (GSE106097), 5 μM SFN-treated SK-UT-1 cells (GSE205777), and 5 mg/ml SFN injected obese mice fed a HFD (GSE181477). To generate the raw count data, the RNA-seq raw data were mapped to the human (or mouse) reference genome (hg19 or mm10) using STAR *via* rsem-calculate-expression in the RSEM package (48). Raw counts and Transcripts per million (TPM)-normalized data were obtained from the results of rsem-calculate-expression. Deposited data are summarized in Table S1.

### RNAseqChef

To develop RNAseqChef, the Shiny package was used to build an interactive web application using the open-source R programming language (https://CRAN.R-project.org/package=shiny). A total of 79 R packages were used to create the core functions of RNAseqChef, as described in the source code (https://doi.org/10.5281/zenodo.7095218). Software and algorithms are summarized in Table S1. The website for RNAseqChef is currently hosted on the shinyapps.io server (https://imeg-ku.shinyapps.io/RNAseqChef/), which may have some limitations such as the capacity of the server and the number of simultaneous connections, and the terms of use as described by shinyapps.io (https://www.rstudio.com/about/shinyapps-terms-use/). To overcome these points, the RNAseqChef Docker image (https://hub.docker.com/r/omicschef/rnaseqchef) has been developed to allow local installation and use on a private computer and network.

### DEG analysis in RNAseqChef

For pair-wise comparison analysis, DEG detection was performed in the “Pair-wise DEG” section that used the DESeq2 package (19) with an FDR cutoff value of 0.01 and 0.05 for *in vitro* and *in vivo* data, respectively. For multiple comparisons of the three groups, DEG detection was performed in the “3 conditions DEG” using the EBSeq package (18) with the following thresholds: fold change >1.25, FDR <0.01, and basemean >1. For the visualization of DEGs, both MA-plot and boxplots were drawn using the ggpubr package (https://CRAN.R-project.org/package=ggpubr), and both volcano plots and scatter plots were drawn using ggplot2 in the tidyverse package (49). In addition, a heat map was drawn using the ComplexHeatmap package (50). For the comparison of DEGs from different datasets, Venn diagram analysis was performed using the Venn package (https://CRAN.R-project.org/package=venn).

### Clustering analysis in RNAseqChef

To evaluate the transcriptomic similarity and heterogeneity of the RNA-seq data, PCA and UMAP were performed using the prcomp function and umap (https://CRAN.R-project.org/package=umap) package, respectively. For gene classification based on NRF2-dependency, k-means clustering was performed using ComplexHeatmap (50).

### Enrichment analysis in RNAseqChef

To conduct functional enrichment analysis, the MSigDB hallmark and DoRothEA regulon gene sets were extracted from the msigdbr (https://CRAN.R-project.org/package=msigdbr) and dorothea packages, respectively (51). Functional enrichment analysis was performed using clusterProfiler (52) with an FDR cut-off of 0.05. The results were visualized *via* dot plots and gene concept-network (cnet) plots using the ggplot2 and enrichplot (https://yulab-smu.top/biomedical-knowledge-mining-book/) packages, respectively (49).

### Quantification and statistical analysis

Statistical analyses were performed using the RNAseqChef software. A pairwise comparison test was performed using the Wald test of DESeq2. Multiple comparison tests were performed using the EBMultiTest function in EBSeq2. Enrichment analysis was performed using Fisher’s exact test. FDR, an adjusted *p* value, was calculated using Benjamini-Hochberg (BH) and Storey’s q-value methods for pairwise DEG detection and enrichment analysis, respectively. Significance was defined as an adjusted *p*-value <0.05, indicated with an asterisk (∗*p* < 0.05, ∗∗*p* < 0.01, ∗∗∗*p* < 0.001).

## Data availability

The RNAseqChef software is available at https://imeg-ku.shinyapps.io/RNAseqChef/ hosted by the shinyapps.io server.

Source code is available at 10.5281/zenodo.7095218.

Docker image is available at https://hub.docker.com/r/omicschef/rnaseqchef.

## Supporting information

This article contains supporting information.

## Conflict of interest

The authors declare that they have no known competing financial interests or personal relationships that could have appeared to influence the work reported in this paper.
