# Supplementary material for: A web-based integrative transcriptome analysis, RNAseqChef, uncovers the cell/tissue type-dependent action of sulforaphane
Source: J Biol Chem. 2023 May 11;299(6):104810. doi: 10.1016/j.jbc.2023.104810 (PMC10267603; doi:10.1016/j.jbc.2023.104810)
Supplement: Supporting information [file mmc1.docx]

**Supporting information**

**A web-based integrative transcriptome analysis, RNAseqChef, uncovers cell/tissue type-dependent action of sulforaphane**

Kan Etoh and Mitsuyoshi Nakao^*^

*Correspondence; [mnakao@gpo.kumamoto-u.ac.jp](mailto:mnakao@gpo.kumamoto-u.ac.jp)

From the Department of Medical Cell Biology, Institute of Molecular Embryology and Genetics, Kumamoto University, Kumamoto, Japan

**
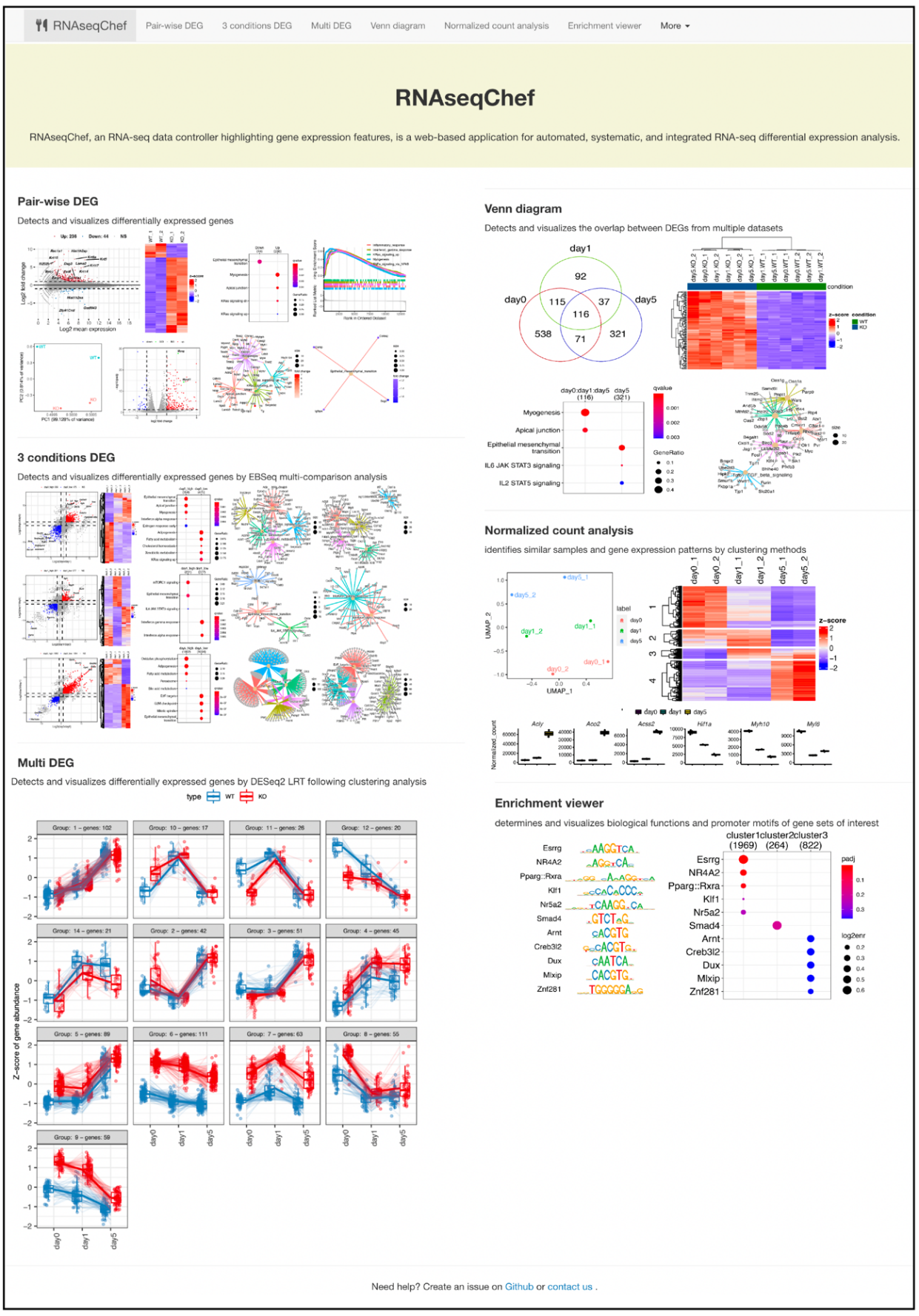
**

**Supplemental Figure S1. Web pages of RNAseqChef, related to Figure 1**

A snapshot of the top page in RNAseqChef.


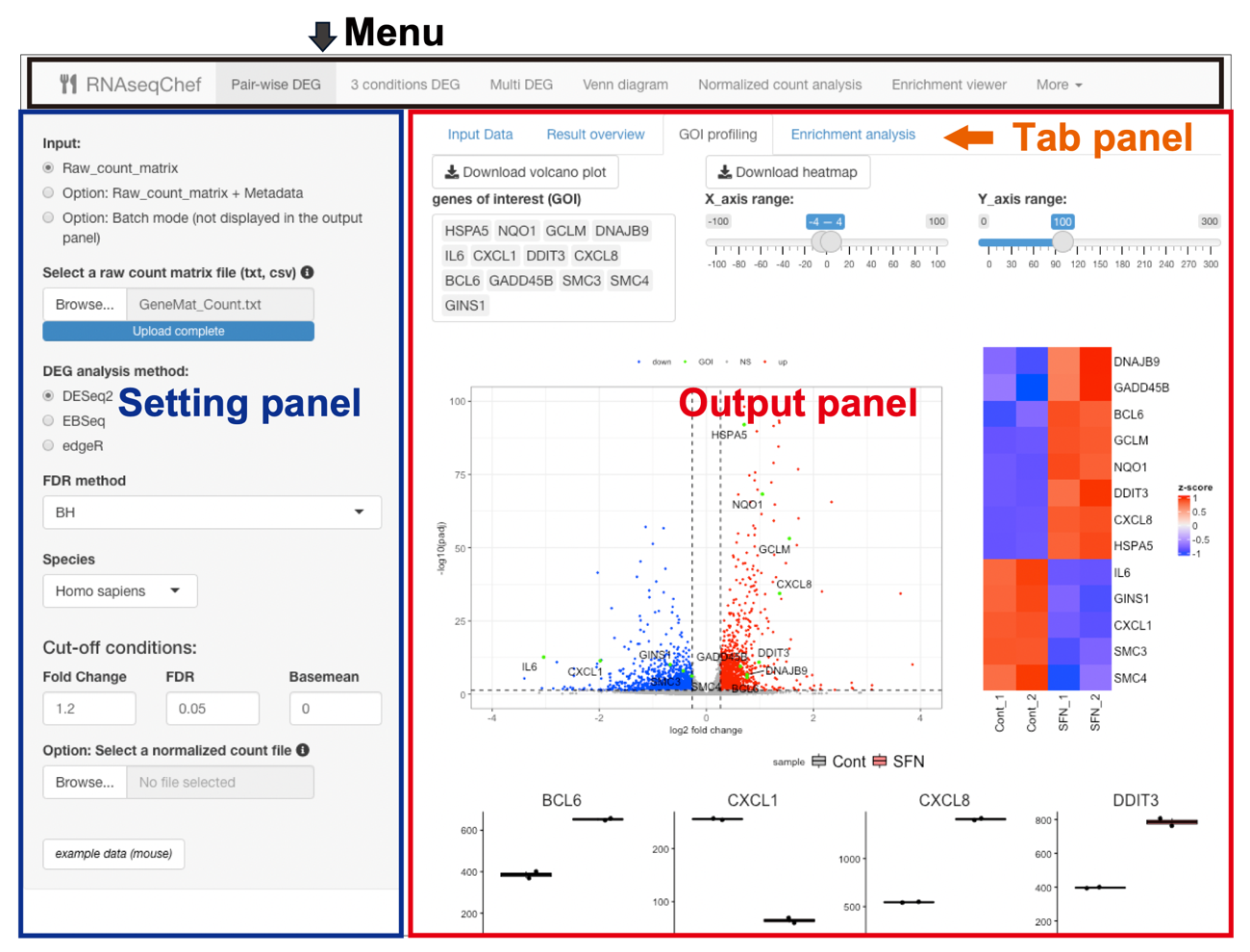


**Supplemental Figure S2. User interface of RNAseqChef, related to Figure 1**

Snapshot of the RNAseqChef webpage. The user interface of RNAseqChef comprises the “Menu”, “Settings”, “Output,” and “Tab” panels. Special bioinformatics skills are not required to operate all functions of RNAseqChef.


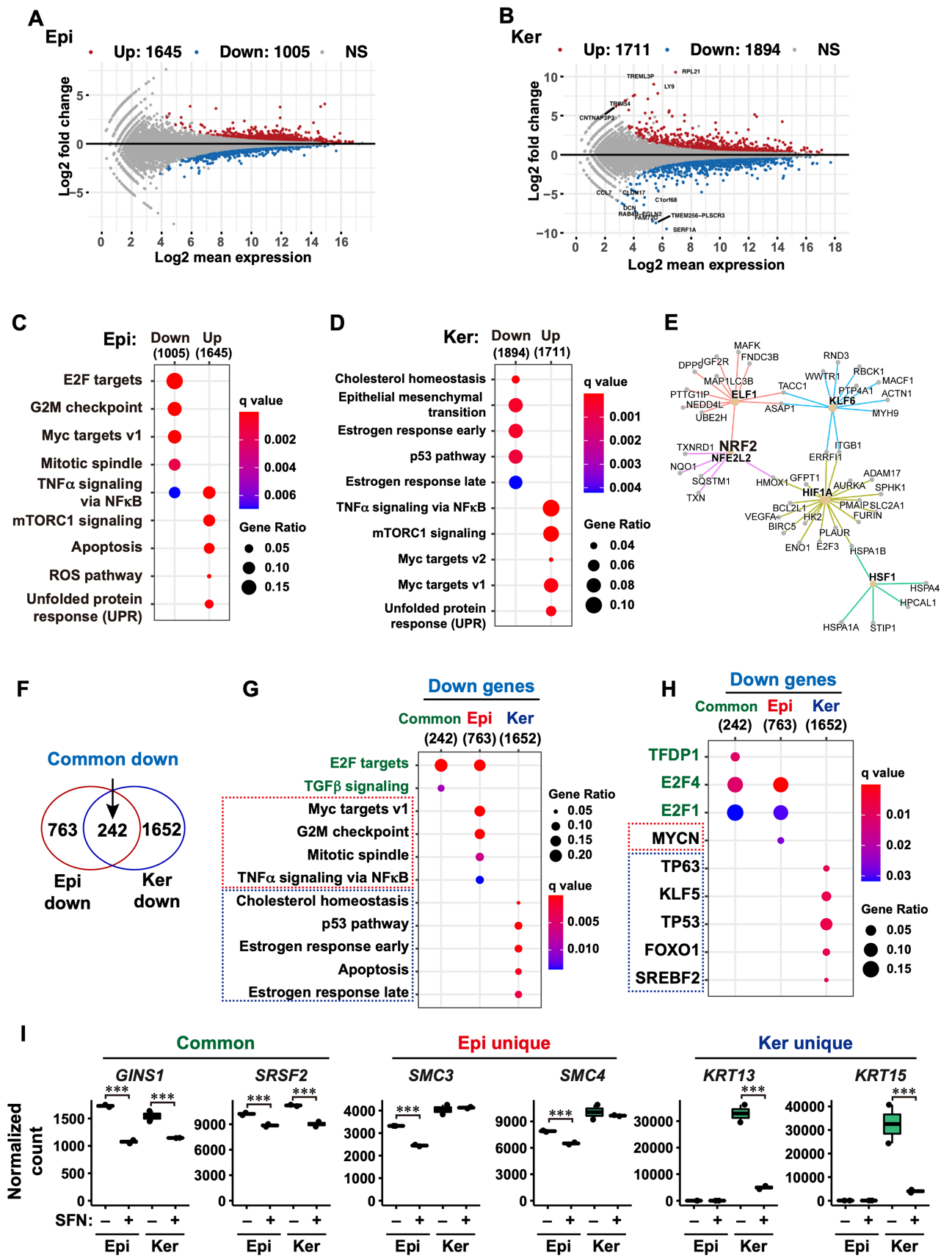


**Supplemental Figure S3. Integrative RNA-seq analysis of SFN-treated Epi and Ker cells *in vitro*, related to Figure 2**

(**A**, **B**) An MA plot of RNA-seq data from epithelial cell (GSE141740) (**A**) and HaCaT keratinocyte (GSE185320) (**B**), which consist of DMSO-treated (Ctrl, n = 2) and 10 μM SFN-treated (SFN, n = 2) samples. FDR < 0.01.

(**C**, **D**) Top-ranked MSigDB Hallmark pathways enriched in the upregulated and downregulated genes in SFN-treated Epi (**C**) and Ker (**D**). FDR < 0.05.

(**E**) Gene-concept network (cnet) plot of the top-ranked transcription factors (TFs) that were enriched in the commonly upregulated genes by SFN, as shown in **Figure 2F**.

(**F**) Venn diagram of genes significantly downregulated by SFN treatment (FDR < 0.01).

(**G**, **H**) Top-ranked functional pathways (**G**) and TFs (**H**) were enriched in 242 commonly downregulated genes (green), 763 Epi uniquely downregulated genes (red), and 1652 Ker uniquely downregulated genes (blue). FDR < 0.05.

(**I**) Normalized expression values of the representative common, Epi-unique, and Ker-unique genes downregulated by SFN. ***, *p* < 0.001.


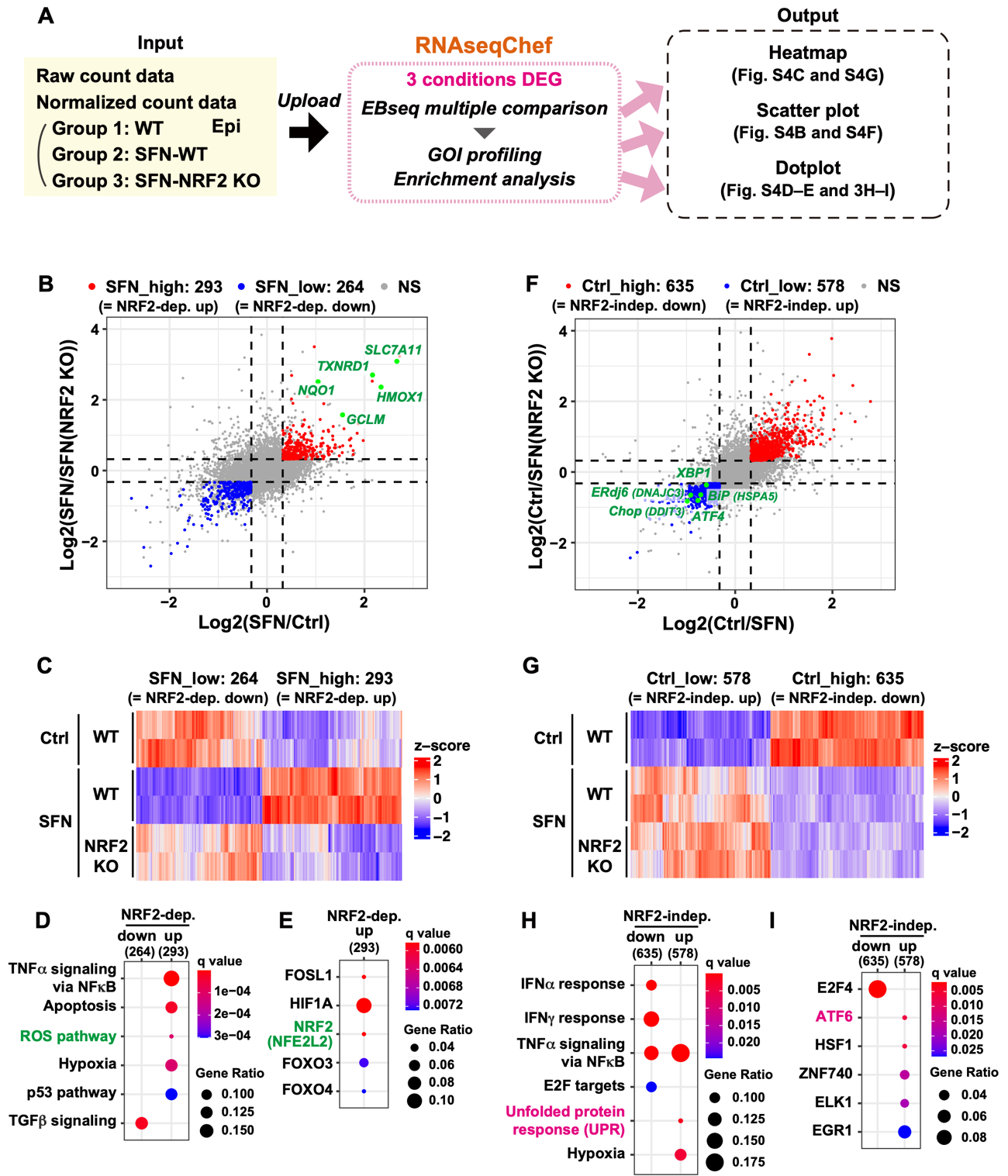


**Supplemental Figure S4. Multiple comparison analysis of three groups using “3 conditions DEG”, related to Figure 3**

(**A**) Schematic representation of multiple comparison analysis of three groups in epithelial cell (Epi) (GSE141740; Ctrl-WT, SFN-WT, and SFN-NRF2 KO, each n = 2), to identify the NRF2-dependent or independent functions of SFN. For DEG detection, EBSeq (FDR < 0.01, fold change > 1.25, basemean > 1) was performed by uploading the raw count files in the “3 conditions DEG” section. Subsequent analysis such as data visualization and enrichment analysis was automatically performed in the tab panels of the same section.

(**B**) Scatter plot of RNA-seq data of three groups. The x- and y-axis show log2 fold change of SFN-WT/Ctrl and SFN-WT/SFN-NRF2 KO, respectively. The red dots indicate SFN_high (e.g. NRF2-dep. up) genes, which are defined as significantly higher expressed genes in SFN-WT than both Ctrl and SFN-NRF2 KO. The blue dots indicate SFN_low (e.g. NRF2-dep. down) genes, which are defined as significantly lower expressed genes in SFN-WT than both Ctrl and SFN-NRF2 KO. The green dots show NRF2-dependent anti-oxidant genes.

(**C**) Heatmap of DEGs shown in **B**. NRF2 KO restored the expression levels of the genes affected by SFN treatment.

(**D**, **E**) Top-ranked functional pathways (**D**) and TFs (**E**) were enriched in 264 downregulated and 293 upregulated genes in an NRF2-dependent manner. FDR < 0.05.

(**F**) Scatter plot of RNA-seq data of three groups. The x- and y-axis show log2 fold change of Ctrl/SFN-WT and Ctrl /SFN-NRF2 KO, respectively. The red dots indicate Ctrl_high (i.e. NRF2-indep. up) genes, which are defined as significantly higher expressed genes in Ctrl than both SFN-WT and SFN-NRF2 KO. The blue dots indicate Ctrl_low (i.e. NRF2-indep. down) genes, which are defined as significantly lower expressed genes in Ctrl than both SFN-WT and SFN-NRF2 KO. The green dots show NRF2-independent UPR genes.

(**G**) Heatmap of DEGs shown in **F**. NRF2 KO could not restore the expression levels of the genes affected by SFN treatment.

(**H**, **I**) Top-ranked functional pathways (**D**) and TFs (**E**) were enriched in 635 downregulated and 578 upregulated genes in an NRF2-independent manner. FDR < 0.05.


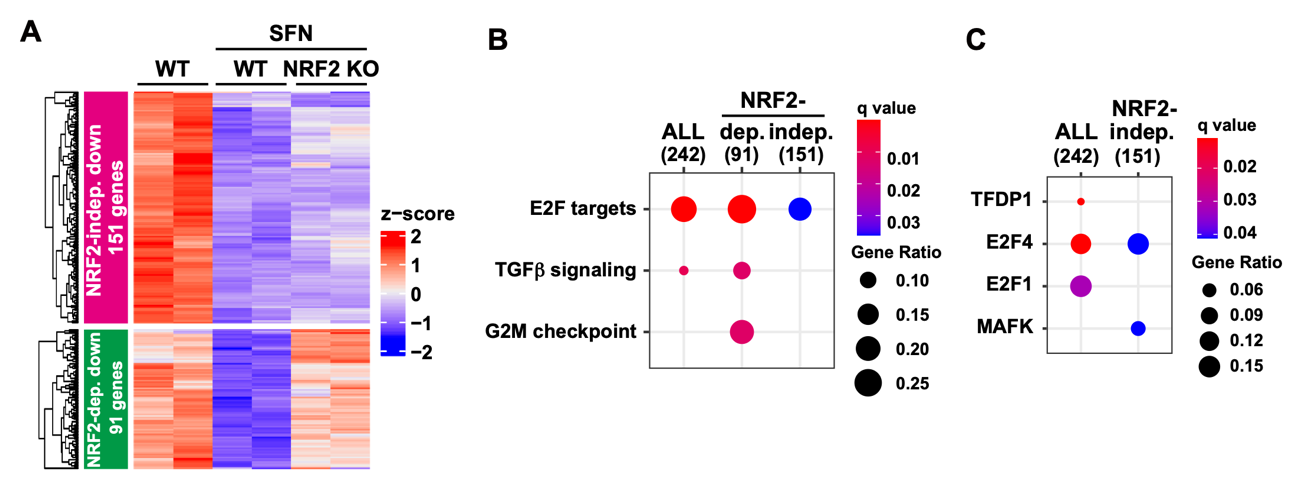


**Supplemental Figure S5. Clustering analysis of genes commonly downregulated by SFN treatment, related to Figure 3**

(**A**) k-means clustering approach separated 242 downregulated genes by SFN treatment (shown in **Supplemental Figure S3F**) into two groups based on NRF2 dependency.

(**B**, **C**) Top-ranked functional pathways (**B**) and TFs (**C**) were enriched in gene sets downregulated by SFN treatment, including 242 ALL genes (sum of the following genes), 91 NRF2-dependent genes, and 151 NRF2-independent genes. Enrichment analysis was performed as same in **Figure 3C**-**D**.


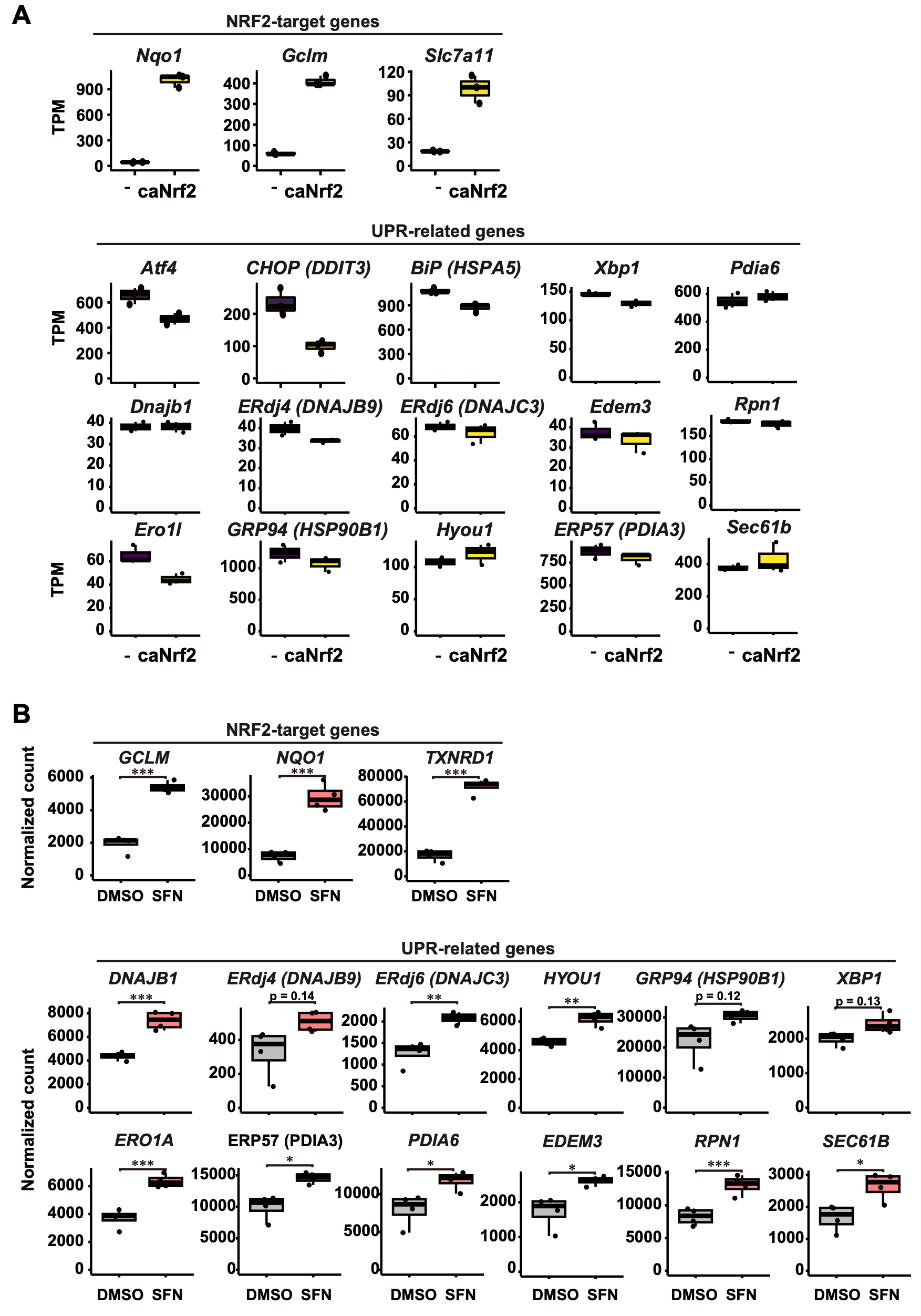


**Supplemental Figure S6. Analysis of constitutively active NRF2 (caNRF2)-expressing cells, related to Figure 3**

(**A**) Transcripts per million (TPM) expression values of representative antioxidant genes (upper) and UPR genes (lower) in untreated Ctrl (-) and caNRF2-expressing cells (GSE106097; each n=3). **, *p* < 0.01, ***, *p* < 0.001.

(**B**) Normalized expression values of the representative antioxidant genes (upper) and UPR genes (lower) in DMSO-treated Ctrl and 5 μM SFN-treated SK-UT-1 cells (GSE205777; each n=4). *, *p* < 0.05, **, *p* < 0.01,***, *p* < 0.001.


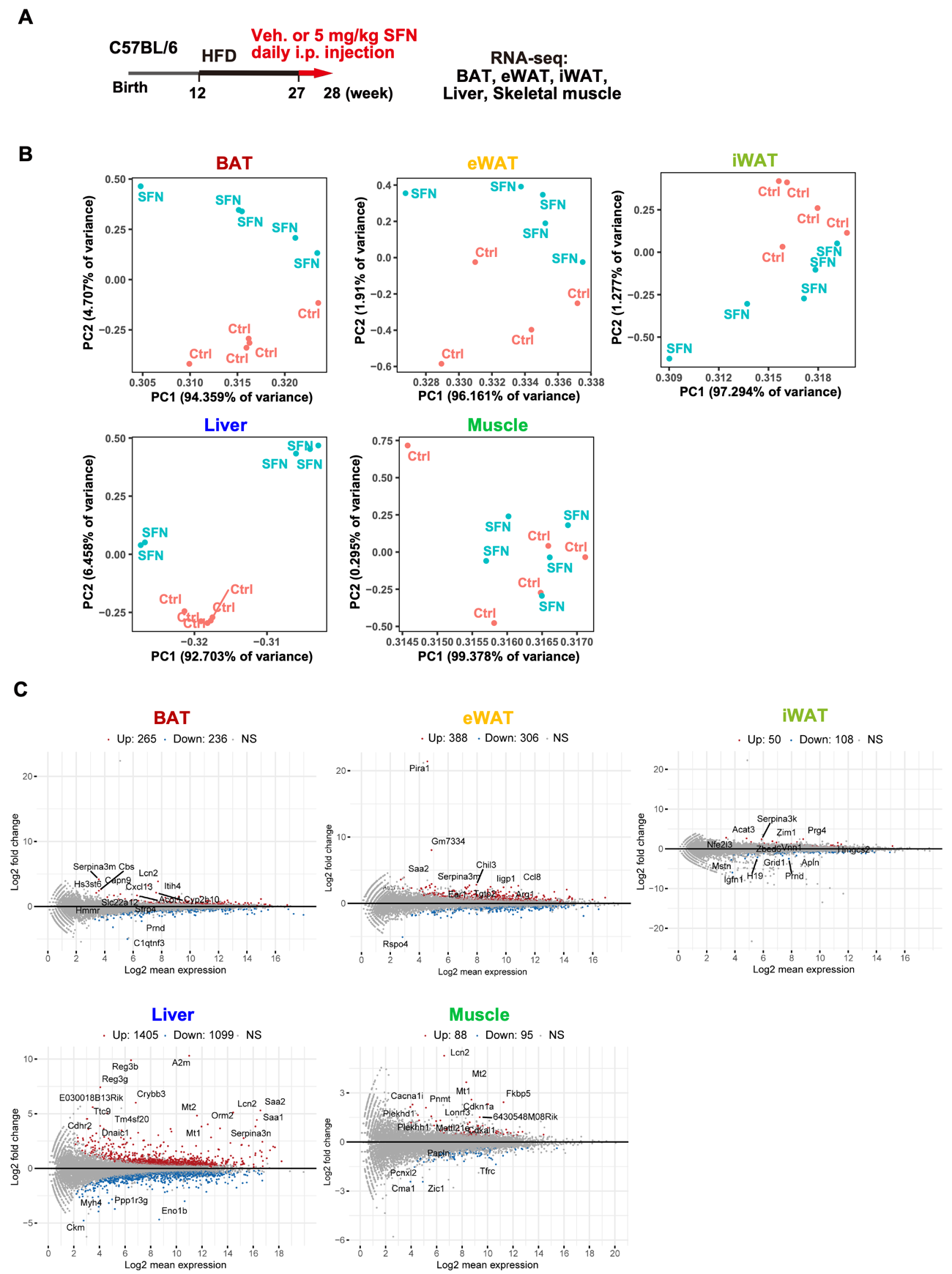


**Supplemental Figure S7. DEG analysis of five metabolic tissues in SFN-treated HFD-fed mice, related to Figure 4**

(**A**) Schematic representation of diet-induced obese model mice (GSE181477). C57BL/6J mice were fed with a high fat diet (HFD; 60% of the calories from fat) for 16 weeks to induce obesity, and were treated with daily intraperitoneal SFN injections (5mg/kg, i.p.) during the last week. After sacrificed, BAT, eWAT, iWAT, liver, and skeletal muscle were collected to perform RNA-seq (Ctrl and SFN-treated, n = 5, except for Ctrl of eWAT (n = 4)).

(**B**) Transcriptome-based PCA of BAT, eWAT, iWAT, liver, and skeletal muscle of SFN-treated HFD-fed mice.

(**C**) An MA plot of RNA-seq data from BAT, eWAT, iWAT, liver and skeletal muscle of SFN-treated HFD-fed mice. FDR < 0.05.

**
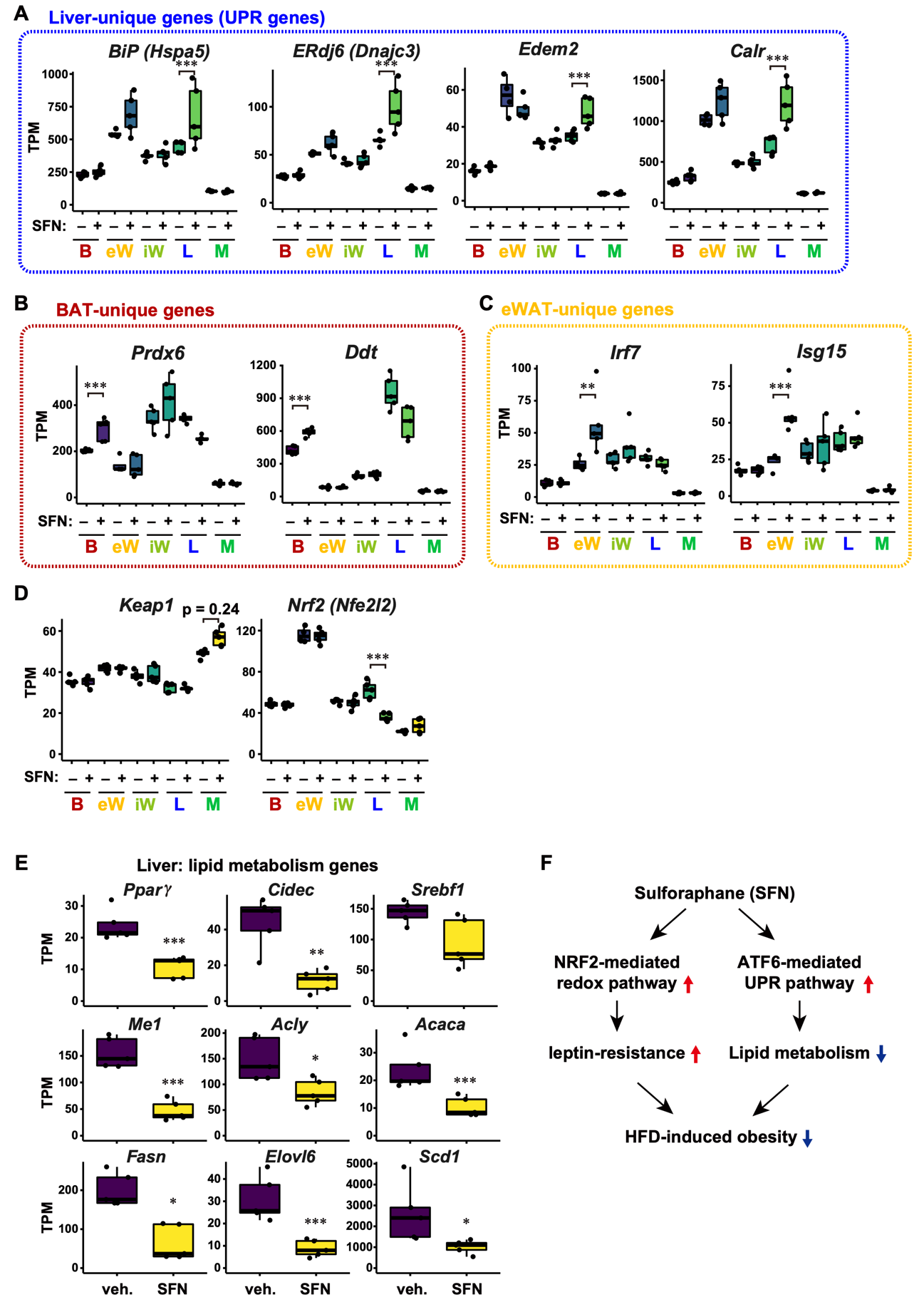
**

**Supplemental Figure S8. Expression profiles of five metabolic tissues in SFN-treated HFD-fed mice, related to Figure 4**

(**A**-**C**) TPM expression values of the representative genes specifically upregulated in liver (**A**), BAT (**B**), and eWAT (**C**).

(**D**) TPM expression values of *Keap1* and *Nrf2* in SFN-treated HFD-fed mice.

(**E**) TPM expression values of the representative lipid metabolism genes in the liver of SFN-treated HFD-fed mice.

(**F**) A proposed model of the roles of SFN under the obese condition. Previous report showed that SFN induces reversing leptin-resistance via NRF2-mediated redox pathway (Çakır et al., 2022). In this study, our data further found that SFN downregulates lipid metabolism in the liver via ATF6-mediated UPR pathway.

**
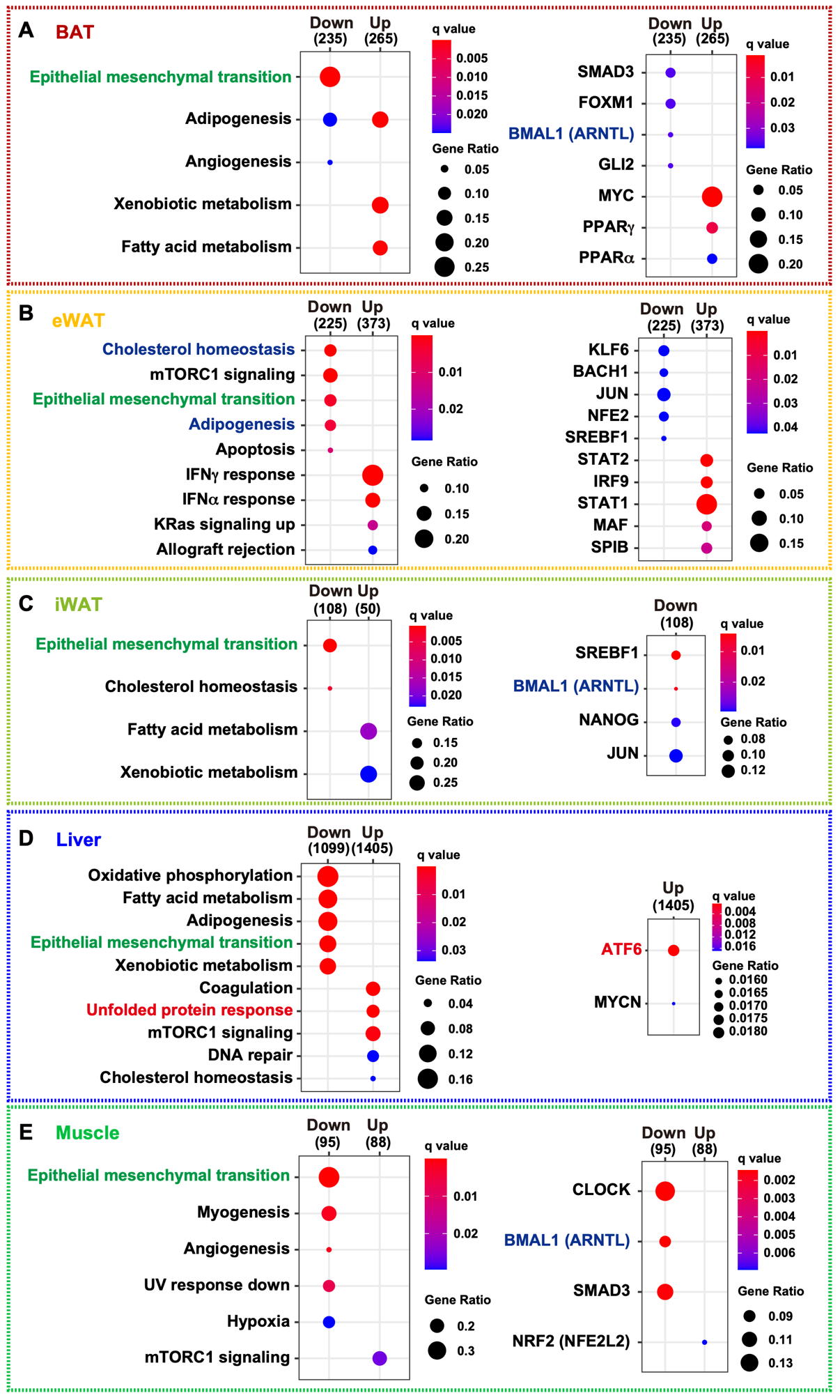
**

**Supplemental Figure S9. Enrichment analysis of DEGs affected by SFN in HFD-fed mice, related to Figures 4 and 5**

(**A**-**E**) Top-ranked functional pathways (left) and TFs (right) were enriched in downregulated and upregulated genes in the BAT (**A**), eWAT (**B**), iWAT (**C**), liver (**D**), and skeletal muscle (**E**) of SFN-treated obese mice. In the liver (**D**), UPR and ATF6 (red) were enriched in 1405 genes upregulated by SFN treatment. “Epithelial mesenchymal transition” (green) was downregulated in all five tissues. In the BAT (**A**), iWAT (**C**), and skeletal muscle (**E**), BMAL1 (blue), a TF for circadian rhythm, was enriched in genes downregulated by SFN treatment. FDR < 0.05.

**
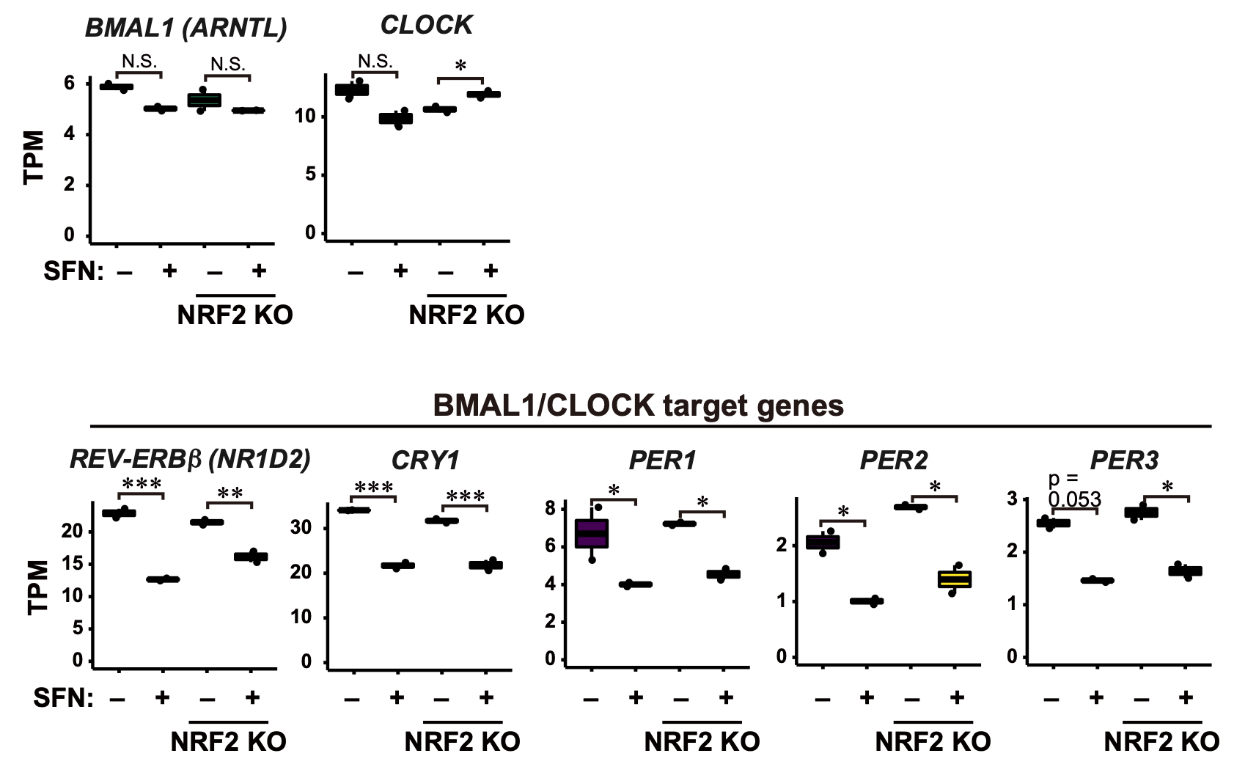
**

**Supplemental Figure S10. Analysis of SFN-treated epithelial cells *in vitro*, related to Figure 5**

TPM expression values of representative circadian rhythm-associated genes in SFN-treated epithelial cells *in vitro* (GSE141740). SFN treatment affected the expression of BMAL1/CLOCK target genes, such as *REV-ERBβ*, *CRY1*, and *PER1/2/3*, in an NRF2-independent manner. *, *p* < 0.05, **, *p* < 0.01, ***, *p* < 0.001.

**
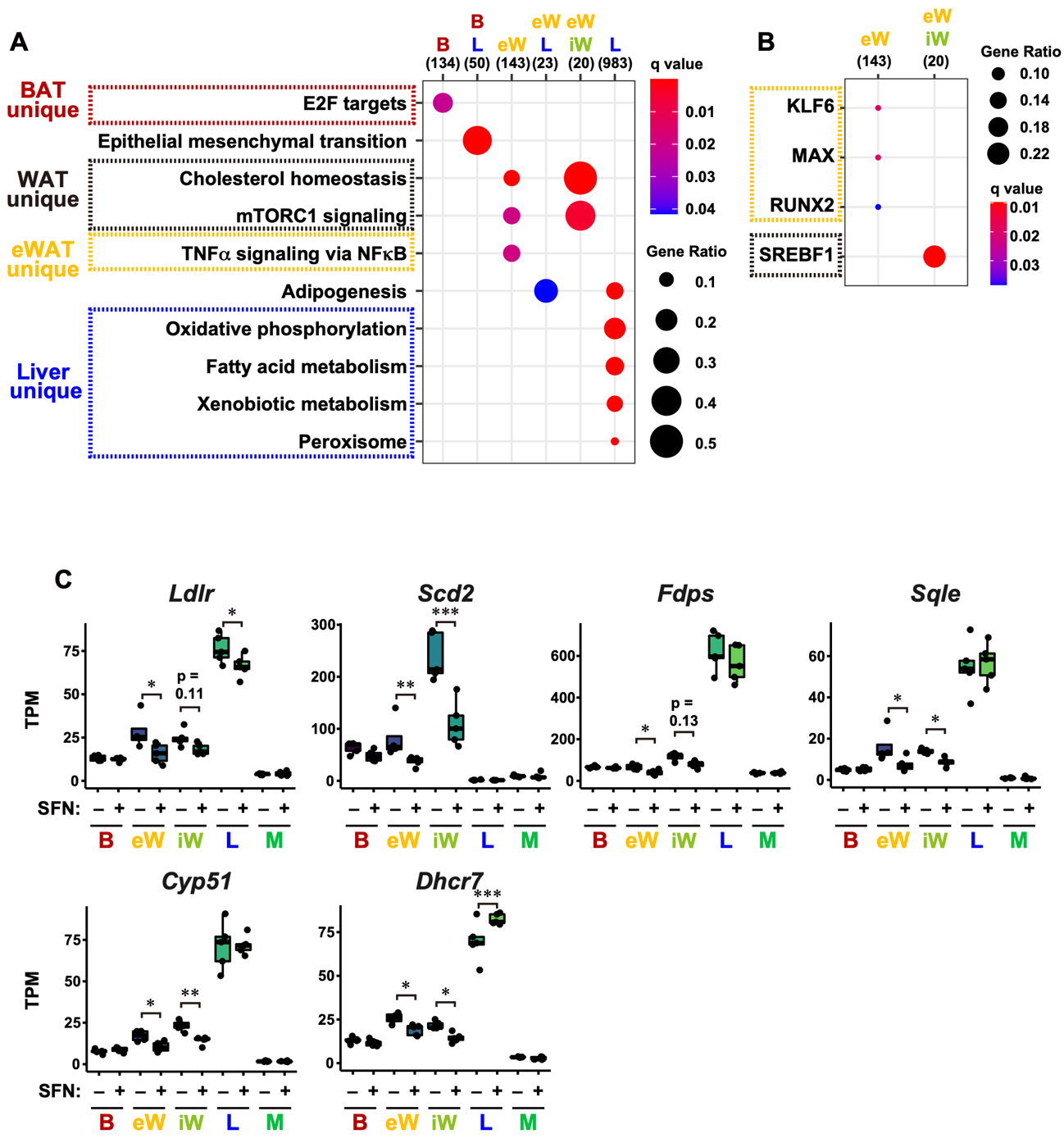
**

**Supplemental Figure S11. Enrichment analysis detects tissue-specific functions of the genes downregulated by SFN in HFD-fed mice, related to Figure 5**

(**A**, **B**) Top-ranked functional pathways (**A**) and TFs (**B**) were enriched in indicated gene sets shown in **Figure 5A**. The intersections that consist of more than 10 genes were tested. Enrichment analysis was performed based on MSigDB hallmark (**A**) and DoRothEA regulon gene set (**B**). The dotted boxes indicate tissue-specific pathways enriched by SFN. FDR < 0.05.

(**C**) TPM expression values of the representative lipid metabolism genes that were downregulated by SFN treatment in eWAT and iWAT. *, *p* < 0.05, **, *p* < 0.01, ***, *p* < 0.001.

**Supplemental Table S1. Deposited data, software and algorithms, related to Methods**

| Resource | Reference | GEO identifier |
| --- | --- | --- |
| Deposited data | | |
| RNA-seq (Epithelial cells) | Lu et al., 2022 | GSE141740 |
| RNA-seq (Keratinocyte) | Li et al., 2022 | GSE185320 |
| RNA-seq (Fibroblasts) | Hiebert et al., 2018 | GSE106097 |
| RNA-seq (SK-UT-1 cells) | Yang et al., 2022 | GSE205777 |
| RNA-seq (obese mice) | Çakır et al., 2022 | GSE181477 |
|  | | |
| Software and algorithms | | |
| RNAseqChef | This study | https://kan-e.shinyapps.io/RNAseqChef/  DOI: 10.5281/zenodo.7095218 |
| R version 4.2.1 | The R Foundation | https://www.r-project.org/ |
| RSEM 1.3.1 | Li and Dewey, 2011 | https://github.com/deweylab/RSEM |
| shiny 1.7.2 | Chang et al. 2022 | https://cran.r-project.org/web/packages/shiny/index.html |
| DESeq2 1.36.0 | Love et al., 2014 | https://bioconductor.org/packages/release/bioc/html/DESeq2.html |
| EBSeq 1.36.0 | Leng et al., 2013 | https://bioconductor.org/packages/release/bioc/html/EBSeq.html |
| clusterProfiler 4.4.4 | Wu et al., 2021 | https://bioconductor.org/packages/release/bioc/html/clusterProfiler.htm |
| dorothea 1.8.0 | Garcia-Alonso et al., 2019 | https://saezlab.github.io/dorothea/ |
| msigdbr 7.5.1 | Dolgalev, 2022 | https://cran.r-project.org/web/packages/msigdbr/index.html |
| venn 1.11 | Dusa, 2022 | https://cran.r-project.org/web/packages/venn/index.html |
| ComplexHeatmap 2.12.1 | Gu et al., 2016 | https://www.bioconductor.org/packages/release/bioc/html/ComplexHeatmap.html |
| ggpubr 0.4.0 | Kassambara, 2020 | https://rpkgs.datanovia.com/ggpubr/ |
| umap 0.2.8 | Konopka, 2022 | https://cran.r-project.org/web/packages/umap/index.html |

**Supplemental Movie Legends**

**Supplemental Movie S1. Overview of all functions of RNAseqChef, related to Figure 1**

Snapshot of all functions of RNAseqChef. Movie S1 consists of two files due to size limitations (S1-1 and S1-2). Please adjust the movie viewing speed appropriately if necessary.

**Supplemental Movie S2. Integrated analysis of the *in vitro* dataset using RNAseqChef, related to Figure 2**

Demonstration of RNAseqChef analysis designed to highlight sulforaphane (SFN)-induced transcriptomic features and their cell type dependency. Raw count data were obtained from public RNA-seq datasets of epithelial cells (Epi; GSE141740) and HaCaT keratinocytes (Ker; GSE185320), under control (Ctrl; DMSO-treated) and 10 μM SFN-treated conditions (each n = 2).

**Supplemental Movie S3. Multiple comparison analysis of three groups using RNAseqChef, related to Figure 3**

Multiple comparison analysis of three groups of epithelial cells (Epi) (GSE GSE141740; Ctrl-WT, SFN-WT, and SFN-NRF2 KO, each n = 2) was performed to identify the NRF2-dependent or independent functions of SFN.
